# Polyphosphate Delays Fibrin Polymerisation and Alters the Mechanical Properties of the Fibrin Network

**DOI:** 10.1160/TH16-01-0062

**Published:** 2016-08-25

**Authors:** Claire S. Whyte, Irina N. Chernysh, Marco M. Domingues, Simon Connell, John W. Weisel, Robert A. S. Ariens, Nicola J. Mutch

**Affiliations:** 1 School of Medicine, Medical Sciences and Nutrition, University of Aberdeen, Aberdeen, UK; 2 Department of Cell & Developmental Biology, University of Pennsylvania, USA; 3 Molecular and Nanoscale Physics Group, School of Physics and Astronomy, University of Leeds, UK; 4 Division of Cardiovascular & Diabetes Research, Faculty of Medicine & Health, University of Leeds, UK

**Keywords:** Fibrin(ogen), polyphosphate, polymerisation

## Abstract

Polyphosphate (polyP) binds to fibrin(ogen) and alters fibrin structure, generating a heterogeneous network composed of ‘knots’ interspersed by large pores. Here we show platelet-derived polyP elicits similar structural changes in fibrin and examine the mechanism by which polyP alters fibrin structure. Polymerisation of fibrinogen with thrombin and CaCl_2_ was studied using spinning disk confocal (SDC) microscopy. PolyP delayed fibrin polymerisation generating shorter protofibrils emanating from a nucleus-type structure. Consistent with this, cascade blue-polyP accumulated in fibrin ‘knots’. Protofibril formation was visualized by atomic force microscopy (AFM) ± polyP. In the presence of polyP abundant monomers of longer length were visualised by AFM, suggesting that polyP binds to monomeric fibrin. Shorter oligomers form in the presence of polyP, consistent with the stunted protofibrils visualised by SDC microscopy. We examined whether these structural changes induced by polyP alter fibrin’s viscoelastic properties by rheometry. PolyP reduced the stiffness (G’) and ability of the fibrin network to deform plastically G’’, but to different extents. Consequently, the relative plastic component (loss tangent (G’’/G’)) was 61 % higher implying that networks containing polyP are less stiff and more plastic. Local rheological measurements, performed using magnetic tweezers, indicate that the fibrin dense knots are stiffer and more plastic, reflecting the heterogeneity of the network. Our data show that polyP impedes fibrin polymerisation, stunting protofibril growth producing ‘knotted’ regions, which are rich in fibrin and polyP. Consequently, the mechanical properties of the fibrin network are altered resulting in clots with overall reduced stiffness and increased ability to deform plastically.

## Introduction

Abnormal clot structure is linked to a number of thrombotic diseases (1–5). The composition of the fibrin network determines the mechanical properties and its stability. Fibrinogen levels are directly associated with fibrin clot structure, with higher concentrations leading to an increase in the number and length of the fibres as well as enhancing fibre diameter. In line with these observations elevated levels of plasma fibrinogen are a risk factor for cardiovascular disease (6). Thrombin concentration has a profound impact on the architecture of a fibrin clot, (7) with high concentrations producing clots composed of thin compactly packed fibres. It is these dense clots that are linked to venous and arterial thromboembolic complications (2, 3, 5, 8–10). The resulting structure of a clot dictates the rate of fibrinolysis. Individually, thin fibres are lysed quicker however, at the network level, clots composed of thick loosely woven fibres are lysed more rapidly (11). Therefore, it is the composition of the fibrin network that influences clot resolution rather than individual fibre diameter.

PolyP is a biomolecule composed of orthophosphate residues (P_i_) linked by phosphoanhydride bonds. PolyP of an average chain length of 60–100 mers is released from the dense granules of stimulated platelets (12, 13). Activation of normal circulating levels of platelets is sufficient to generate concentrations of around 1–3 μM polyP in whole blood (12, 14). However, in the milieu of a thrombus local polyP concentrations are likely to be several orders of magnitude higher due to the substantial accumulation of platelets. PolyP is both pro-inflammatory and pro-coagulant *in vivo* (13, 15) and modulates haemostasis at a number of points in the cascade (reviewed in (16)) including binding fibrin(ogen) and altering the structure of the resulting clot (17, 18). Inhibition of polyP inhibits thrombin generation at arterial shear rates and fibrin generation at both venous and arterial shear rates (19) and polyP inhibitors have been recently proposed as novel antithrombotic agents (20, 21). We have shown that fibrin networks produced in the presence of polyP are very heterogeneous with dense ‘knotted’ regions interspersed by pores (18). A consequence of this altered structure is attenuation of tissue plasminogen activator (tPA) and plasminogen binding to fibrin thereby down-regulating tPA (16)-mediated fibrinolysis in the presence of polyP (18). The regulatory role of polyP in haemostasis has been proposed to act to promote rapid resolution of injury upon activation of platelets by enhancing coagulation, whilst inhibiting fibrinolysis to allow repair (14).

Here we sought to determine the molecular mechanisms underlying the structural changes observed in fibrin clots formed in the presence of polyP of approximately the size range of that found in platelets (60–100 mer) (13). We investigated changes in early fibrin polymerisation in the presence of polyP in real-time using spinning disk confocal (SDC) microscopy and at high resolution using atomic force microscopy. PolyP delayed fibrin polymerisation producing stunted protofibrils which moved with a reduced velocity. Rheological measurements indicate that clots formed with polyP exhibit altered mechanical properties which may alter their ability to respond to shear stress *in vivo.*

## Materials and methods

### Materials

Plasminogen free human fibrinogen (Fib 1) and Glu-plasminogen were purchased from Enzyme Research Laboratories (Swansea, UK). Alexa-fluor 488 (AF488) conjugated fibrinogen from human plasma, Cascade Blue™ ethylenediamine, trisodium salt were from Molecular Probes (Leiden, The Netherlands) and 4.5 μm superparamagnetic beads (Dynabeads M-450 Epoxy) were from Life Technologies (Paisley, UK). Thrombin from human plasma (T1063) and polyphosphate (type 65; polyP) were purchased from Sigma-Aldrich (Dorset, UK). Capillary tubes for magnetic tweezers were 0.5 mm in diameter and 5 cm in length (VitroCom; Mountain Lakes, NJ, USA). 1-ethyl-3-[3-(dimethylamino)propyl]carbodiimide (EDAC), Slide-A-Lyzer dialysis cassettes G2 (2K MWCO 3 ml) and DyLight 550™ (DL-550) antibody labelling kit were purchased from Thermo Scientific (Rockford, IL, USA). PolyP of an average chain length of 70 phosphate monomers, used for the cascade blue labelling, was a kind gift from Dr Thomas Staffel BK Giulini GmbH (Ludwigshafen, Germany). Platelet-derived polyP was extracted from outdated apheresis platelets as described (22). PolyP concentrations are expressed as monomer concentrations throughout (monomer formula NaPO_3_). Where indicated, thrombin and fibrinogen concentrations were altered due to the specific requirements and constraints of the experimental procedures. Unless otherwise stated all reactions were carried out with human plasminogen-free fibrinogen (1.5 mg/ml) ± polyP (328 μM) with thrombin (0.25 U/ml) and calcium chloride (5 mM) in TBS (50 mM tris, 100 mM NaCl pH 7.4).

### Fibrin polymerisation measurements

Changes in turbidity were monitored by taking measurements every 30 s at 340 nm for 60 minutes (min) at 37°C using a BioTek ELX808 microplate plate reader. SDC microscopy studies of fibrin polymerisation were carried out as previously described (23) with the following modifications. Fibrinogen (1.62 mg/ml, 7 % as AF488-fibrinogen) was polymerised by the addition of thrombin (0.25 U/ml) and CaCl_2_ (5 mM) ± polyP. Polymerisation reactions took place in Ibidi μ slides VI_0.4_ (Ibidi, Martiensried, Germany) with a channel height of 400 μm. A stack of 30 images was acquired every 15 seconds (s) for 45 min at 37°C on a UltraVIEW Vox 3D live cell imaging system with a plan Apo VC ×60/1.40 NA oil objective OFN 25 DIC N2 using Velocity software (Perkin Elmer, Waltham, MA, USA). The velocity of the moving fibrin structures was calculated by measuring the distance travelled between subsequent images of 3 representative structures from each data set using Image J software (National Institutes of Health, Bethesda, MD, US).

### Clot rheometry

The viscoelastic properties of the overall fibrin network were measured on an AR G2 Rheometer (TA Instruments; New Castle, DE, USA). Measurements were taken during polymerisation at 37°C every 18 s for 80 min using a 40 mm parallel plate, gap of 220 mm, strain of 2 % and frequency of 5 rad/s. The elastic modulus, G’, gives information about the energy stored during deformation translated into clot stiffness. The loss modulus, G’’, gives information about energy loss during deformation translated into the viscous component of the clot. The loss tangent *tan*δ = *G”/G’* was also calculated.

In addition, an in-house built magnetic tweezers system was used to examine local microrheology in fibrin clots (24) by applying a magnetic force over a 4.5 μm superparamagnetic bead and measuring its displacement in the clot. Clots, formed in the presence of superparamagnetic beads by addition of thrombin (0.25 U/ml) to fibrinogen (0.5 mg/ml) and CaCl_2_ (2.5 mM), ± polyP, were immediately transferred to a thin capillary tube and allowed to polymerise for 3 hours (h). An electromagnetic force (40 pN) was applied using four electromagnets mounted on top of an inverted Olympus IX-71 microscope (Olympus; Southend, UK) and connected to Kepco BOP 20–5M amplifiers (Kpeco Inc.; Flushing, NY, USA) controlled using LabView software (National Instruments; Newbury, UK). The position of the magnetic beads was tracked using CCD camera and measurements made, avoiding capillary walls where the clot was not densely packed. Both elastic and viscous modulus of the fibrin clots were calculated from the time-dependent compliance in order to get frequency dependent moduli (25). The compliance was calculated as the ratio of the time-dependent shear strain (time-dependent bead displacement) to the magnitude of the constant stress (force applied). In each clot the displacement of 10 particles (in different areas of the clot) were measured and each clot was studied in triplicate. The values presented in this paper were obtained at 0.1 Hz.

### Confocal microscopy

Cascade blue ethylenediamine (CB)-labelled polyP was prepared as described (26) and used to visualise its localisation within the fibrin network. Briefly, polyP (1 mg/ml) was incubated overnight at 37°C with Cascade Blue ethylenediamine (1 mM), CaCl_2_ (1 mM), EDAC (100 mM), and 2-(N-morpholino)ethanesulfonic acid (100 mM), pH 6.5. CB-polyP adducts were purified using Slide-A-Lyzer dialysis cassettes. Clots were formed ± CB-polyP (328 μM) or platelet-derived polyP by polymerising fibrinogen (0.87 mg/ml, containing 9 % labelled with DL550-fibrinogen or AF488-fibrinogen) with thrombin (0.25 U/ml) and CaCl_2_ (5 mM).

To visualise the localisation of the superparamagnetic beads, clots were prepared as for the magnetic tweezers microrheology experiments in μ-slide VI_0.4_ (Ibidi) with the inclusion of AF488-fibrinogen (0.12 mg/ml). Z-stack images (0.45 or 0.5 μm slices) were taken on a Zeiss 710 laser scanning confocal microscope with a 63 × 1.40 oil immersion objective using Zeiss Zen 2012 software.

### Atomic force microscopy

Protofibril formation was visualised by atomic force microscopy (AFM) by clotting fibrinogen (0.5 mg/ml) with thrombin (1 U/ml) and CaCl_2_ ± polyP. Polymerisation was allowed to proceed for 40 s before stopping the reaction by diluting in TBS and placing an aliquot on freshly cleaved MgCl_2_ mica pre-treated surface for imaging in air using a Bruker Multimode AFM and SNL-A Scanasyst probe.

### Statistical analysis

Statistical analysis was performed in GraphPad Prism® 5.04 using one-way analysis of variance or two-way analysis of variance with Bonferroni post-hoc test or an unpaired Student’s t-test (2-tailed). P < 0.05 was considered to be significant. Changes in rates of velocity (μm^2^/s) for fibrin polymerisation measured by SDC microscopy were determined by best fit to a centered sixth order polynomial quadratic in GraphPad Prism® 5.04 and used to calculate fold differences in velocity.

## Results

### PolyP accumulates in fibrin dense ‘knots’

**Figure 1:**
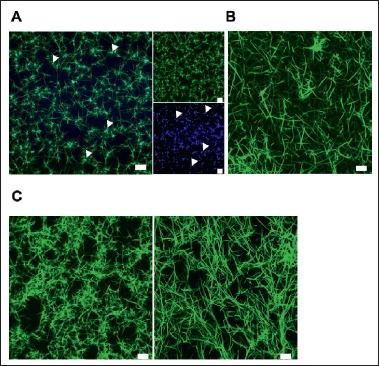
**Visualisation of polyP with fibrin network.** Clots were formed ± CB-polyP or platelet-derived polyP (328 μM) by polymerising fibrinogen (0.87 mg/ml, with 9 % labelled with DL550-fibrinogen or AF488-fibrinogen) with thrombin (0.25 U/ml) and CaCl_2_ (5 mM). A) Render of Z-stack representative image polyP-containing clot with fibrinogen (green, top right panel) and polyP (blue, bottom right panel) and the merged image (left panel). Arrows indicate the presence of polyP in fibrin dense regions. B) Control clot formed in the absence of polyP. C) Clot containing platelet-derived polyP (left) and control clot (right) for comparison. Images were collected using a LSM 710 confocal microscope. Representative image from n = 3, scale bar = 10 μm.

The localisation of CB-polyP within the clot was determined using confocal microscopy. CB-polyP accumulated in the knotted fibrin dense regions with a reduced fluorescent signal detected along the fibrin fibres that emanate from the ‘knots’ (▶ Figure 1A, see also Suppl. Video I, available online at www.thrombosis-online.com). A control clot formed in the absence of CB-polyP is shown for comparison (▶ Figure 1B). PolyP exists in various polymer lengths, with platelet-derived polyP ranging between 60 and 100 phosphate residues (12, 13). Inclusion of polyP extracted from human platelets during fibrin polymerisation mirrored the structural changes observed with synthetic polyP, with fibrin dense knots interspersed by large pores (▶ Figure 1C).

### PolyP alters fibrin polymerisation

**Figure 2:**
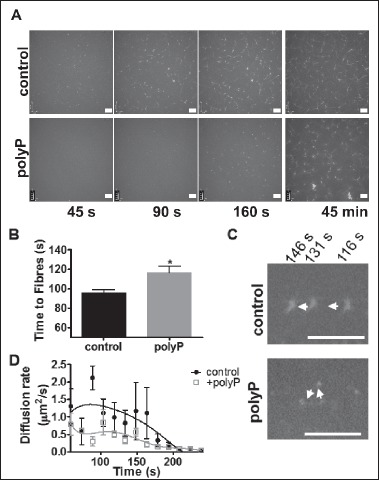
**PolyP alters fibrin polymerisation.** Fibrinogen (1.62 mg/ml, 7 % as AF488-fibrinogen) was polymerised by the addition of thrombin (0.25 U/ml) and CaCl_2_ (5 mM) ± polyP (328 μM) and monitored by SDC microscopy. A stack of 30 images was acquired every 15 s for 45 min at 37°C. A) Time course images showing delayed polymerisation in the presence of polyP and the stabilised clots at 45 min. B) The time at which fibres were first visualised. C) Overlaid images from representative structures ± polyP at three different time points. Arrows indicated direction of movement, scale bar = 10 μm. D) The velocity of the fibrin structures was calculated by measuring the distance travelled between subsequent images of three representative structures from each data set using Image J software. Data are expressed as mean ± SEM, n = 4.

Our previous observations showed that polyP reduced the maximum turbidity of polyP-containing clots indicating an altered fibrin network, which we confirmed by confocal and scanning electron microscopy (18). There was no detectable change in the lag time, however, change in turbidity is a crude measurement which does not allow for the real time study of the very early polymerisation events. Here we use SDC microscopy to visualise the dynamic process occurring during the first few minutes of polymerisation. Initially, in control clots, small very fast moving structures were observed that gradually form thicker protofibrils before stabilizing into a fibrin network (Suppl. Video II, available online at www.thrombosis-online. com). The presence of polyP resulted in smaller structures and delayed the time at which the first fibres were first observed by 21 s (p <0.05) (▶ Figure 2A, B). Measurements of velocity were taken by tracking the movement of the same structure over time (▶ Figure 2C). Quantification of the velocity of the fibrin structures revealed a decrease in movement over time in both control and polyP clots (▶ Figure 2D). This reduction in velocity over time is likely attributed to the increased size of the structures, due to elongation and lateral aggregation of fibres with time (23). Clots containing polyP demonstrated a 2.4-fold (p <0.05) reduction in velocity compared to control clots (▶ Figure 2C, D). Stabilised clots (▶ Figure 2A, right panel) polymerised in the presence of polyP displayed the same heterogeneous knotted structures as previously observed (▶ Figure 1; Suppl. Video I, available online at www.thrombosis-online.com).

### Protofibrils are stunted during polymerisation with polyP

**Figure 3:**
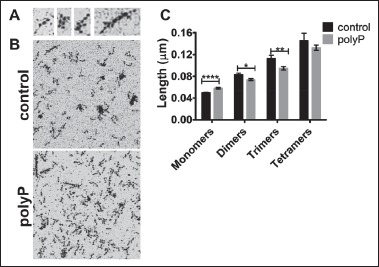
**Protofibril formation visualised using AFM.** Fibrinogen (0.5 mg/ml) was polymerised for 40 s by addition of thrombin (1 U/ml) and CaCl_2_ (5 mM) ± polyP and the reaction stopped by diluting. Samples were imaged using a Bruker Multimode AFM and SNL-A Scanasyst probe (air). A) Representative images of a monomer, dimer, trimer and tetramer structure. Clearly distinguishable are the central E regions, D regions and in the monomers the αC regions. B) Representative images from control and polyP containing reactions. Images show a 1 μm^2^ area. C) Calculation of structure length using Nanoscope analysis software. Twelve fibrin structures representing monomers, dimers, trimers and tetramers were measured from three separate images.

SDC microscopy is capable of detecting protofibrils of a length of 0.5 μm and greater (23). We further addressed the effects of polyP on early protofibril formation using AFM, which is a very high resolution technique that allows detection in the nanometer range. ▶ Figure 3A shows representative images of monomers, dimers, trimers and tetramers. The structure of the fibrin monomers including the αC regions were clearly visible at this high resolution (▶ Figure 3A). After 40 s of polymerisation in the presence of polyP more abundant fibrin monomers are observed (▶ Figure 3B), indicative of slower fibrin polymerisation, consistent with the observations using SDC microscopy. The monomeric fibrin structures (exhibiting clearly visible D-E-D regions) were approximately 8.8 nm longer in the presence of polyP compared to the control (58.4 ± 1.4 nm vs 49.6 ± 0.6 nm, p <0.0001) (▶ Figure 3C). This elongation likely indicates direct binding of polyP to fibrin monomers, consistent with our previous demonstration of interaction with soluble fibrin (18). Binding of polyP to fibrin monomers subsequently attenuated the size of the resulting dimers and trimers (▶ Figure 3C). Larger fibrin structures are visible in the control clot compared to the polyP containing clot indicative of more advanced protofibril formation. These AFM observations suggest that the association of polyP and fibrin mechanically alters oligomer formation during early fibrin polymerisation steps.

### PolyP modulates the rheological properties of the clot

**Figure 4:**
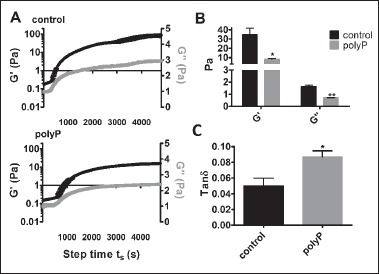
**Rheological properties of the fibrin network.** Measurements were taken during polymerisation of fibrinogen (1.5 mg/ml) ± polyP (328 μM) by the addition of thrombin (0.25 U/ml) and CaCl_2_ (5 mM) on a rheometer at 37°C every 18 s for 80 min. Shown are plots (A) and histograms (B) of the average storage (G’) and loss (G’’) moduli and (C) the loss tangent ([Formula: *tan*δ = *G‘‘/G‘*]). * P <0.05, ** P <0.01 compared with control clots. Data are expressed as mean ± SEM, n = 3.

We next addressed whether the structural changes in fibrin observed in the presence of polyP alter the rheological properties of the clot. This was first measured in a rheometer during the polymerisation process. Clots containing polyP were 4-fold less stiff than control clots (8.3 ± 0.8 Pa vs 34.7 ± 6.9 Pa, p < 0.05) as indicated by their reduced storage modulus (G’), which is a measure of the stored energy and represents the elastic component (▶ Figure 4A, B). The loss modulus (G’’), which represents the energy dissipated as heat, was 2-fold higher in the control clots compared to those containing polyP (1.6 ± 0.1 vs. 0.7 ± 0.01, p < 0.01; ▶ Figure 4A, B). As the change in the storage modulus was greater, the loss tangent (G’’/G’) for polyP containing clots was 57 % (p < 0.05) higher in the presence of the polyP, indicating an increase in the relative plastic component (▶ Figure 4C). The lag time was prolonged in clots containing polyP, consistent with the delayed polymerisation observed by SDC microscopy. These results imply that in the presence of polyP the overall fibrin network is less stiff and more likely to deform plastically.

**Figure 5:**
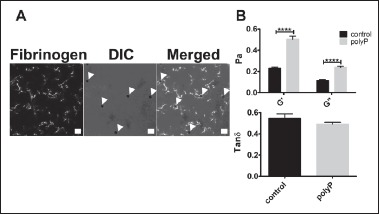
**Magnetic tweezers measurements of the local rheological properties.** Fibrin clots were formed by addition of thrombin (0.25 U/ml) to fibrinogen (0.5 mg/ml) and CaCl_2_ (2.5 mM), ± polyP (328 μM) in the presence of superparamagnetic beads. A) Localisation of the superparamagnetic beads was visualized within the clots with the inclusion of AF488-fibrinogen (19 % of total fibrinogen) on a Zeiss 710 laser scanning confocal microscope. DIC = differential interference contrast. Arrows indicate supraparamagnetic beads, scale bar = 10 μm. B) An electromagnetic force (40 pN) was applied and the position of the magnetic beads tracked using CCD camera. Displacement of 10 particles per clot were measured and each clot was studied in triplicate. The values presented were obtained at frequency 0.1Hz. **** P <0.0001, compared with control clots. Data are expressed as mean ± SEM, n = 3.

The microscale viscoelastic properties of fully polymerised cross-linked fibrin clots were further investigated using magnetic tweezers. In this method clots are polymerised in the presence of supraparamagnetic beads which are trapped within the fibrin network. Due to the heterogeneous nature of polyP-containing clots, we analysed the location of the beads by confocal microscopy and found them to localise within the knotted fibrin regions (▶ Figure 5A). The tightly knotted fibrin areas in polyP-containing clots were 2-fold stiffer than homogeneous control clots (G’ 0.50 ± 0.03 vs 0.23 ± 0.01 Pa, p < 0.0001) (▶ Figure 5B). Inclusion of polyP also increased the loss modulus 2-fold (G’’ 0.24 ± 0.01 vs 0.12 ± 0.01 Pa, p < 0.0001) and as a result there was no significant difference in the tan delta values. This suggests that the fibrin dense knots, which accrue high concentrations of polyP, are stiffer in nature. Taken together, these different viscoelastic measurements reflect the highly heterogeneous nature of the fibrin network formed in the presence of polyP.

## Discussion

Our observations (18) and others (17) have previously illustrated that polyP modulates the structural properties of a fibrin clot. Here we demonstrate that these structural alterations in the fibrin network observed with synthetic polyP are mirrored with natural polyP that has been isolated from human platelets. The heterogeneity of the fibrin network in the presence of polyP can be attributed to early events during clot development. We visualise fibrin formation in real-time using SDC microscopy (23) and show that polyP significantly delays polymerisation of fibrin. High resolution AFM microscopy reveals binding of polyP to fibrin monomers resulting in shorter protofibrils that exhibit reduced velocity during polymerisation. These changes in polymerisation generate clots composed of fibrin dense aggregates, which contain a polyP-rich core, and are interspersed by large porous regions. The extremely heterogeneous nature of polyP-containing clots is echoed in their viscoelastic properties, with distinct differences in the microscale and overall clot stiffness.

PolyP binds to fibrinogen and soluble fibrin (18) and is incorporated into clots during polymerisation (17). PolyP stored in platelet dense granules has a defined chain length of 60–10 phosphate residues (12, 13) Here, we demonstrate that the structural changes observed with synthetic polyP are representative of those induced by natural form of polyP isolated from human platelets. Using AFM we visualise the association of polyP with fibrin monomers, illustrated by the longer length of these microstructures. This binding results in accumulation of polyP within fibrin dense regions, suggesting it acts as a nucleus for fibrin formation. In bacteria, polyP functions as a chaperone to stabilise cytoplasmic proteins by binding to unfolded proteins and preventing the formation of protein aggregates (27).

Precipitation of polyP into spherical nanoparticles in the presence of 5 mM CaCl_2_ has recently been described (28). These nanoparticles had more potent activity towards the contact system. We have previously shown that polyP has the most pronounced effect on fibrinolysis at a CaCl_2_ concentration of 2.5–5 mM (18) and in these studies have used 5 mM throughout. We also found that the polyP used in this study was visualised as a spherical form by AFM (data not shown), suggesting it functions as a polyP nanoparticle. During fibrin polymerisation polyP may act as a surface that attracts fibrinogen, creating dense areas of fibrin. This initial network forms the scaffold for deposition of subsequent fibrinogen molecules (29, 30). Therefore the localisation of polyP within these early stages of polymerisation may act as a blueprint for the resulting fibrin network.

The rate of polymerisation has a direct impact on the resulting fibrin network structure. During the lag phase there is rapid movement of fibrin structures with formation of the initial scaffold corresponding to the gel point (30). As the turbidity increases the scaffold stabilises allowing branch point formation and lateral growth of fibres (30). In general, the turbidity of fibrin gels is proportional to the average cross-sectional area of the fibres (31). However, in the presence of polyP, the resulting heterogeneous clot structure provides an example of a clot where the turbidity is not proportional to the average cross-sectional area of the fibres. Inclusion of polyP delayed polymerisation observed by SDC microscopy producing stunted fibrin structures with reduced mobility. Consistent with the SDC microscopy, AFM revealed a reduction in oligomer length, which is directly linked to the rate of polymerisation (32).

The viscoelastic properties of the fibrin network are closely related to its mechanical stability. Here the rheological properties of polyP-containing clots, quantified during polymerisation, demonstrated a reduction in both the storage and loss moduli. The decrease in storage modulus was greater, resulting in an increased loss tangent indicating that polyP-containing clots are less stiff with an increased plastic component. These characteristics may seem conflicting; however, they likely reflect the heterogeneous nature of these clots, with the porous loose regions accounting for the reduction in stiffness. We also assessed the local rheological properties of fully polymerised and cross-linked clots using magnetic tweezers. In these measurements the supraparamagnetic beads were directly incorporated into the knotted regions of fibrin that are rich in polyP. Using this technique we found an increase in the storage modulus with polyP, indicative of an increased stiffness. These observations are in agreement with thromboelastography measurements performed on fully polymerised clots (17). These differences, while conflicting reflect the ongoing changes that occur in a fibrin network when it transitions from the polymerisation stage to a fully formed scaffold (30). The addition, removal or inhibition of factor XIII does not alter the effects of polyP (17, 18). Therefore, the changes in the fibrin network are not explained by differences in cross-linking. Changes in the viscoelastic properties of a clot will affect how it responds to forces such as blood flow. Clots with a reduced storage modulus are more likely to deform irreversibly and as the plastic component of these clots increases, induced deformation is likely to be permanent (33). The stiffness of the clot remains constant over a range of different rates of stress (33) therefore within platelet-rich areas of a clot, with high local concentrations of polyP, this would reduce the overall stiffness and modulate how the clot reacts to different stresses such as clot retraction and shear rate.

**What is known about this topic?**

- PolyP binds to fibrin(ogen).
- PolyP alters the fibrin network structure forming clots with fibrin aggregates interspersed by large pores.

**What does this paper add?**

- PolyP delays fibrin polymerisation and stunts protofibril growth.
- PolyP is localised in the dense fibrin knots.
- Overall clots containing polyP are less stiff and more likely to deform plastically.

The αC region of fibrinogen compromises the terminal two thirds of the carboxyl terminal of the Aα chain. During conversion of fibrinogen to fibrin the αC regions dissociate from the central E domain allowing intermolecular interactions and enhancing lateral aggregation (34–36). Interestingly, clots formed with fibrinogen truncated at Aα residue 251, show a reduction in stiffness and more plastic deformation (37). There is a net positive charge on the αC regions which could favour interaction with negatively charged polymers such as polyP (38). The αC regions are the most unfolded part of fibrin(ogen) and in view of the fact that polyP acts as chaperone to unfolded proteins in bacteria (27), it is conceivable that it interacts with polyP in a similar manner. PolyP increased the length of fibrin monomers which could be due to the αC region extending beyond the D-region, resulting in longer structures. During formation of larger structures the αC region has to extend laterally rather than longitudinally to allow D-D interactions which could be enhanced by polyP, perhaps resulting in the observed shortened oligomers. It is interesting to speculate that by binding to this region polyP alters the structural and rheological properties of the fibrin network, but further work is necessary to characterise the exact binding site of polyP on fibrinogen.

Detection of abnormal changes to clot structure has the potential as a biomarker of thrombotic disease. Recently, clot fractal dimension *(d_f_)* has been proposed as a test for abnormal clot microstructure and predictor of recurrent VTE (39). Changes in the fibrin network translate into alterations in the susceptibility to fibrinolysis. The resulting heterogeneous network with polyP has a reduced permeability and the rate of fibrinolysis by tPA is reduced due to impaired binding of tPA and plasminogen to partially degraded fibrin (18). Interestingly, the binding sites for tPA and plasminogen on fibrin are also concentrated in the αC regions suggesting a possible mechanism by which polyP attenuates binding of these proteins.

In conclusion, we have shown that platelet-derived polyP elicits the same structural changes in fibrin as previously described for synthetic polyP. We found that polyP modulated the early polymerisation events of fibrin formation by binding directly to fibrin(ogen), thereby altering oligomer formation, producing stunted fibrin structures that move with reduced velocity. PolyP acts as a nucleus for dense accumulations of fibrin which are interspersed by a looser porous network. The heterogeneous nature of the network is conveyed in its viscoelastic properties producing a clot with reduced stiffness and increased ability to deform plastically, but with local micro-pockets of increased stiffness in areas where polyP is concentrated on fibrin.

## Supplementary Material

Suppl. Video I: Suppl. Video I – PolyP accumulates in fibrin dense knotted regions Clots were formed ± CB-polyP (328 μM) by polymerizing fibrinogen (0.87 mg/ml, containing 9 % labelled with DL550-fibrinogen) with thrombin (0.25 U/ml) and CaCl2 (5 mM). Shown is a render of Z-stack images collected using a LSM 710 confocal microscope. Fibrin(ogen) is shown in green and CB-polyP in blue. Representative video from n = 3.

Suppl. Video II: Suppl. Video II – Fibrin polymerization. Fibrinogen (1.62 mg/ml, containing 7 % as AF488-fibrinogen) was polymerized by the addition of thrombin (0.25 U/ml) and CaCl2 (5 mM) ± polyP65 (328 μM) and monitored by SDC microscopy. A stack of 30 images was acquired every 15 s for 45 min at 37 °C. Video shows images captured over the first 6 min from onset of polymerization. Representative images of n = 4.
